# Germ-Free Swiss Webster Mice on a High-Fat Diet Develop Obesity, Hyperglycemia, and Dyslipidemia

**DOI:** 10.3390/microorganisms8040520

**Published:** 2020-04-05

**Authors:** Isabelle E. Logan, Gerd Bobe, Cristobal L. Miranda, Stephany Vasquez-Perez, Jaewoo Choi, Malcolm B. Lowry, Thomas J. Sharpton, Andrey Morgun, Claudia S. Maier, Jan F. Stevens, Natalia Shulzhenko, Adrian F. Gombart

**Affiliations:** 1 Department of Biochemistry and Biophysics, Linus Pauling Institute, Oregon State University, Corvallis, OR 97331, USA; logani@oregonstate.edu; 2 Department of Animal Sciences, Linus Pauling Institute, Oregon State University, Corvallis, OR 97331, USA; gerd.bobe@oregonstate.edu; 3 Department of Pharmaceutical Sciences, Linus Pauling Institute, Oregon State University, Corvallis, OR 97331, USA; Cristobal.Miranda@oregonstate.edu (C.L.M.); fred.stevens@oregonstate.edu (J.F.S.); 4 Carlson College of Veterinary Medicine, Corvallis, OR 97331, USA; vasquest@oregonstate.edu (S.V.-P.); Natalia.Shulzhenko@oregonstate.edu (N.S.); 5 Linus Pauling Institute, Oregon State University, Corvallis, OR 97331, USA; jaewoo.choi@oregonstate.edu; 6 Department of Microbiology, Linus Pauling Institute, Oregon State University, Corvallis, OR 97331, USA; Malcolm.Lowry@oregonstate.edu; 7 Department of Microbiology, Oregon State University, Corvallis, OR 97331, USA; Thomas.Sharpton@oregonstate.edu; 8 Department of Statistics, Oregon State University, Corvallis, OR 97331, USA; 9 Department of Pharmaceutical Sciences, Oregon State University, Corvallis, OR 97331, USA; andriy.morgun@oregonstate.edu; 10 Department of Chemistry, Oregon State University, Corvallis, OR 97331, USA; claudia.maier@oregonstate.edu; 11 Linus Pauling Institute, Department of Biochemistry and Biophysics, Oregon State University, Corvallis, OR 97331, USA

**Keywords:** gut bacteria, germ free, metabolic syndrome, type 2 diabetes, diet-induced obesity

## Abstract

A calorie-dense diet is a well-established risk factor for obesity and metabolic syndrome (MetS), whereas the role of the intestinal microbiota (IMB) in the development of diet-induced obesity (DIO) is not completely understood. To test the hypothesis that Swiss Webster (Tac:SW) mice can develop characteristics of DIO and MetS in the absence of the IMB, we fed conventional (CV) and germ-free (GF) male Tac:SW mice either a low-fat diet (LFD; 10% fat derived calories) or a high-fat diet (HFD; 60% fat derived calories) for 10 weeks. The HFD increased feed conversion and body weight in GF mice independent of the increase associated with the microbiota in CV mice. In contrast to CV mice, GF mice did not decrease feed intake on the HFD and possessed heavier fat pads. The HFD caused hyperglycemia, hyperinsulinemia, and impaired glucose absorption in GF mice independent of the increase associated with the microbiota in CV mice. A HFD also elevated plasma LDL-cholesterol and increased hepatic triacylglycerol, free fatty acids, and ceramides in all mice, whereas hypertriglyceridemia and increased hepatic medium and long-chain acylcarnitines were only observed in CV mice. Therefore, GF male Tac:SW mice developed several detrimental effects of obesity and MetS from a high-fat, calorie dense diet.

## 1. Introduction

Diet-induced obesity (DIO) impairs glucose and lipid homeostasis, increases chronic systemic inflammation, causes hypertension, and is a primary risk factor for hepatic steatosis, type II diabetes mellitus (T2DM), and cardiovascular disease (CVD) [1,2,3,4,5]. Clinically, individuals are diagnosed with metabolic syndrome (MetS) if they possess three or more of the following indicators: abdominal obesity, hypertension, insulin resistance, high serum triacylglycerol (TAG), and low high-density lipoprotein cholesterol (HDL-C) concentrations. MetS afflicts over 34% of the population in the Americas, Australia, and Europe [6,7,8]. Palatable, calorie-dense diets such as the high-fat and high-sucrose Western diet are a major cause of DIO in humans, and similar diets induce obesity in mouse models [9,10,11,12]. These diets are rich in simple digestible sugars, saturated lipids, or both, and possess a high fractional feed conversion. Moreover, when fed these diets, mice do not sufficiently decrease feed consumption to maintain their caloric energy balance [13]. In mice, abdominal obesity, insulin resistance, and other indicators of MetS develop with DIO [14,15,16]. 

Increasingly, studies suggest alterations to the intestinal microbiota (IMB) contribute to the development of obesity, MetS, T2DM, CVD, and non-alcoholic fatty liver disease (NAFLD) [17]. The IMB plays an important role in energy harvest and obesity-associated inflammation [11,18,19,20,21]. Thus, diet-induced alterations in IMB composition combined with an increase in sedentary lifestyles may explain how the IMB contributes to obesity [22,23]. Comparison of conventional (CV) and germ-free (GF) mice provides valuable information about the role of the IMB in immune system development and behavior of the host [24,25]. GF mice also provide the foundation for gnotobiotic research, allowing the colonization of the gut with individual or defined populations of microbes [26]. To understand the role of the IMB in obesity and MetS, it is important to compare the effects of diet and treatment in vivo between GF and CV animals. Table 1 summarizes several prior studies using different mouse strains and diets. Inbred C57BL/6J mice are well established for DIO studies and offer a wide variety of genetic knockouts [27,28]; however, susceptibility of GF C57BL/6J mice to DIO remains unclear. Previous studies show mixed results, indicating that GF C57BL/6J are partly protected against lard induced DIO and white adipose tissue inflammation [10,11,29,30,31]. It is possible to attribute these mixed results to dietary sucrose levels, with low sucrose lard-based high fat diets (HFDs) increasing the effects of microbiota-induced obesity [32,33]. For example, conventionalized adult, male C57BL/6J mice developed obesity on a high-fat, high-sucrose, calorie dense diet, whereas GF mice were protected due to an increase in β-oxidation [10,31]. In this case, male GF C57BL/6J mice gained less weight on an HFD than conventionalized C57BL/6J due to lower feed intake and absorption, specifically of lipids [10,31]. In another study, GF C57BL/6J mice showed a dramatic weight increase on a lower-sucrose lard-based HFD, whereas these mice were protected from DIO on a fish oil-based HFD, suggesting both sucrose content and fat source (lard is rich in saturated lipids, fish oil in polyunsaturated lipids) affect weight gain [33]. The effects of diet composition were further investigated for the inbred C57BL/6N strain [34]. Here, evidence supported a role for cholesterol in resistance to DIO in GF mice, as cholesterol affected the crosstalk between the IMB and host metabolism [34]. This became particularly evident when GF C57BL/6N mice fed a low-sucrose cholesterol-free palm oil-based HFD experienced a weight increase on par with their CV counterparts [34]. However, GF mice were protected from weight gain on a low-sucrose high-cholesterol lard-based HFD, unlike CV C57BL/6N mice receiving the same diet [34]. Finally, inbred GF male C3H not only gained more body weight and body fat, but also showed lower energy expenditure than their CV counterparts when fed a low-sucrose lard-based HFD, thus exhibiting characteristics of human DIO; however, energy intake was not reported [32]. These studies suggest both diet composition and mouse strain play a role in DIO in the GF state.

All Swiss Webster mice are derived from nine mice imported to the United States from Switzerland in 1926 by Clara J. Lynch [35]. In 1932, Lynch sent mice to Leslie Webster, who subsequently sent mice to other academic and commercial breeders [36]. Swiss Webster mice are albino and genetically heterogeneous when outbred [36]. Therefore, not all Swiss Webster mice are alike, as mice obtained from The Jackson Laboratory are inbred, whereas mice from Taconic Biosciences (used in this research) are outbred [36]. Hence, it is important to state the source of the mouse strain used. All Swiss Webster mice carry a mutation in the *Cd5* gene, which they share with inbred strains such as C57BL/6J, and Taconic Swiss Webster (Tac:SW) mice carry the recessive mutation, *Pde6b^rd1^*, which leads to retinal degradation [37]. However, neither one of these mutations affects DIO. Swiss Webster mice differ from C57BL/6J, in that they do not carry the *Nnt^C57BL/6J^* mutation, which is responsible for impaired glucose clearance in the C57BL/6J strain [38]. Despite this, Swiss Webster mice do exhibit a genetic susceptibility to diabetes, as spontaneous development of obesity-associated polyuric, polydipsic, glucosuric, and hyperglycemic symptoms were observed previously [39]. Subsequently, Swiss Webster mice are reasonably prone to DIO, and the research community uses commercially available GF Swiss Webster mice for IMB transplant studies [36,39,40,41,42,43,44,45,46,47]. Bäckhed and colleagues recently developed a simplified human IMB model using GF Swiss Webster mice that holds promise in the study of diet–host–microbiota interactions in relation to obesity and MetS [40]. However, to study these interactions in relation to DIO, it is necessary to determine the role that the IMB play in the development of obesity and MetS in Swiss Webster mice. Therefore, we hypothesized that Tac:SW mice can develop characteristics of DIO and MetS in the absence of the IMB. To test this hypothesis, we used a 2 × 2 experimental design, and fed outbred CV and GF male Tac:SW mice a diet containing either 10% or 60% fat-derived calories for 10 weeks. We show that the absence of the IMB did not prevent most of the detrimental effects of a low-sucrose, low-cholesterol, lard-based HFD in the development of obesity and MetS.

## 2. Materials and Methods 

### 2.1. Experimental Design

Experimental animal procedures were performed in accordance with Animal Care and Use Protocol 5053 approved 28 March 2019 by the Institutional Animal Care and Usage Committee of Oregon State University (OSU, Corvallis, Oregon, USA). Sex differences in the development of DIO in mice are well established and documented, especially in C57BL/6 mice [48]. Female mice are generally considered protected from DIO [49,50]. Therefore, we purchased male GF and CV Tac:SW mice at 8–10 weeks of age (Taconic Biosciences, Rensselaer, NY, USA). GF Tac:SW mice were housed in gnotobiotic isolators and CV mice in specific pathogen free conditions at the Laboratory Animal Resource Center at OSU under a standard 12-h light cycle and 22 ± 1 °C ambient temperature. After acclimation, mice were randomly assigned to either a low-fat diet (LFD control) or an HFD (*n* = 10 per group for CV, and *n* = 11 per group for GF mice) and housed individually. Autoclaved water was supplied to the mice ad libitum. The GF status of mice was routinely confirmed by culturing and PCR analysis of feces using universal primers amplifying the 16S rRNA, as described previously [51]. Feed intake and body weight were measured weekly, and feed conversion was calculated using the Formula (1):

(1) $$\mathrm{Feed}\;\mathrm{Conversion}\;(\%)=\frac{\mathrm{Weight}\;\mathrm{Gain}\;\left(g\right)}{\mathrm{Feed}\;\mathrm{Consumption}\;\left(g\right)}\times 100\%$$

After 10 weeks of feeding, the mice were anesthetized, and blood was collected for plasma by cardiac puncture. Subsequently, the mice were euthanized by cervical dislocation and various tissue collected, flash frozen in liquid nitrogen, and stored at −80 °C. Livers and fat pads were weighed before freezing.

### 2.2. Diets

Mice were fed either an LFD (containing 10%, 70%, and 20% total calories from fat, carbohydrate, and protein, respectively) or an HFD (containing 60%, 20%, and 20% total calories from fat, carbohydrate, and protein, respectively) as pellets (Dyets, Inc., Bethlehem, PA, USA) ad libitum. The diet was irradiated at a dose of 50 kGy to sterilize it (Radiation Center at OSU, Corvallis, OR, USA). The diet composition is summarized in Table 2. The majority of calories in the LFD control were derived from cornstarch, whereas this changed to lard for the HFD.

### 2.3. Measurement of Blood Metabolic Profiles

Concentrations of blood glucose and plasma concentrations of TAG, and leptin were determined as described previously, and plasma insulin was determined using a mouse insulin ELISA kit (Alpco Diagnostics, Salem, NH, USA) [52]. The plasma HDL-C concentration was determined using the MaxDiscovery™ HDL-C Cholesterol Assay Kit (Bio Scientific Corporation, Austin, TX, USA). The plasma LDL-C concentration was determined using the Wako L-type LDL-C assay kit (Wako Diagnostics, Mountain View, CA, USA). 

### 2.4. Glucose Tolerance Test

A glucose tolerance test (GTT) was performed after 10 weeks of feeding. Following a 6-h fast, mice were injected intraperitoneally with a glucose solution (CV, 1 g/kg body weight; GF, 2 g/kg body weight). GF mice received a higher concentration of glucose to ensure an increase in blood glucose concentrations. Blood taken from the tail vein was sampled using a hand-held glucometer at 0, 15, 30, 60, and 120 min, as described previously [52].

### 2.5. Lipidomics and Acylcarnitine Quantification

For lipidomics analysis, liver tissue (approximately 50 mg) was ground using a Precellys 24 homogenizer (Bertin Corp, Rockville, MD, USA) with 1.4 mm zirconium oxide beads (OMNI International, Kennesaw, GA, USA) in ice-cold methylene chloride:isopropanol:methanol (25:10:65, *v*/*v*/*v* +BHT (0.05%); 50 µg/mL). The volume of solvent was proportional to the tissue weight (10 μL solvent per 1 mg). The homogenate was incubated at −20 °C for 1 h. The mixture was centrifuged at 13,000× *g* at 4 °C for 10 min. A 30 µL aliquot from the supernatant was added to a mass spec vial, after which extraction solvent (165 µL) and SPLASH^®^ LIPIDOMIX^®^ Mass Spec Standard (5 µL, Avanti Polar Lipids Inc., Alabaster, AL, USA) were added. An aliquot (3 μL) of the supernatant was subjected to UPLC-QTOF analysis on a 5600 TripleTOF instrument (Sciex, Concord, ON, Canada) as described previously [53].

For acylated and free carnitine quantification, liver tissue (approximately 50 mg) was ground using a Precellys 24 homogenizer (Bertin Corp, Rockville, MD, USA) with 1.4 mm zirconium oxide beads (OMNI International, Kennesaw, GA, USA) in ice cold methanol:water (80:20, *v*/*v*), using a volume of solvent proportional to the tissue weight (10 μL solvent per 1 mg). The homogenate was incubated at −20 °C for 1 h. The mixture was centrifuged at 13,000× *g* at 4 °C for 10 min. The supernatant was collected and lyophilized using an SC110A SpeedVac Plus coupled to an RVT400 refrigerated vapor trap (Thermo Fisher Scientific, Inc., Waltham, MA, USA). The dry extracts were then reconstituted in 200 µL of acetonitrile:water (1:1, *v/v*), vortexed for 30 sec, and centrifuged at 4 °C for 5 min at 13,000 rpm. An aliquot of the supernatant was subjected to UPLC-QTOF analysis on a 5600 TripleTOF instrument (Sciex, Concord, ON, Canada). Data analysis was performed using Metaboanalyst 4.0 software (http://www.metaboanalyst.ca/) [54].

### 2.6. Statistical Analysis

Statistical analyses of continuous data were performed using the method of least squares to fit general linear models in SAS (SAS Institute, Cary, NC, USA) using a 2 × 2 design with diet, microbiota, and their interaction as fixed effects, whereas for categorical data, Fisher’s exact test was used. All statistical tests were two-sided, and statistical significance was set at *p* ≤ 0.05. 

## 3. Results

### 3.1. Conventional and Germ-Free Tac:SW Mice Develop Obesity on the High Fat Diet

**Figure 1:**
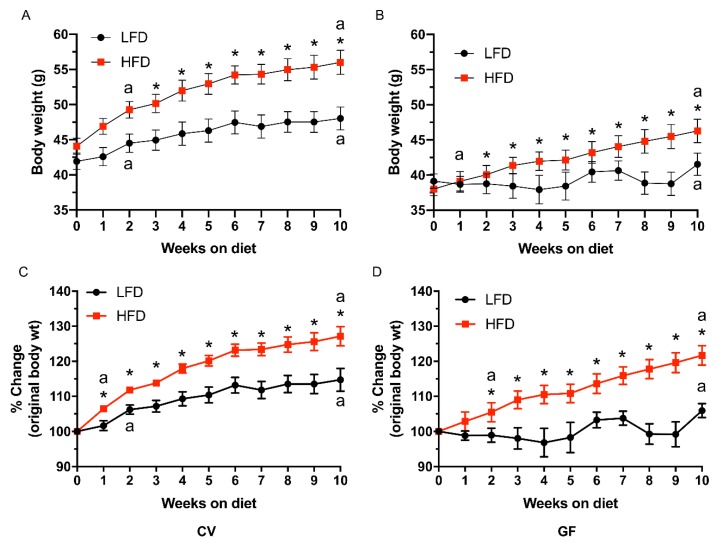
The HFD increases body weight in both CV (*n* = 10; panels **A**,**C**) and GF (*n* = 11; panels **B**,**D**) male Tac:SW mice. Absolute body weight changes are shown in grams (panels **A**,**B**), or as percent change from original body weight (panels **C**,**D**). Values are expressed as the mean ± SEM. An “a” denotes statistically significant increases in body weight, compared to baseline body weight at week 0, and are only shown at the start and end of statistical significance. An asterisk denotes statistically significant diet difference within CV and within GF mice, when comparing the effects of the HFD on weight gain to the LFD, at *p* ≤. 0.05. Abbreviations: CV, conventional; GF, germ-free; HFD, high fat diet; LFD, low fat diet; Tac:SW; Taconic Swiss Webster.

The HFD increased body weight in both CV and GF mice (Figure 1 and Table 3). The effect of the HFD on body weight gain was immediate with statistically significant differences beginning week 2 and 1 in CV and GF mice, respectively (Figure 1). Even for CV mice on the LFD, a statistically significant increase in body weight was observed by week 2 (Figure 1A,C). In contrast, GF mice on the LFD did not gain body weight until week 10 (Figure 1B,D). In summary, the IMB is not required for DIO in male Tac:SW mice, and the IMB allow CV male Tac:SW mice to gain weight even on the LFD. 

Body weight gain resulted from greater feed conversion on the HFD (an average increase from 1.52% on LFD (CV and GF combined) to 4.10% on HFD (CV and GF combined); results are for main diet effect and not shown in Table 3). The increase in feed conversion and body weight observed with the HFD was independent of the increase associated with the presence of the IMB (Table 3). Throughout the study, CV mice were heavier than GF mice. Presence of the IMB modified the effect of the HFD on feed consumption (*p* = 0.03) and fat pads (*p* < 0.001; Table 3). In contrast to CV mice, GF mice did not decrease feed intake on the HFD (*p* = 0.48), resulting in significantly heavier fat pads (2.77 g on the HFD versus 0.77 g on the LFD; *p* < 0.001).

**Figure 2:**
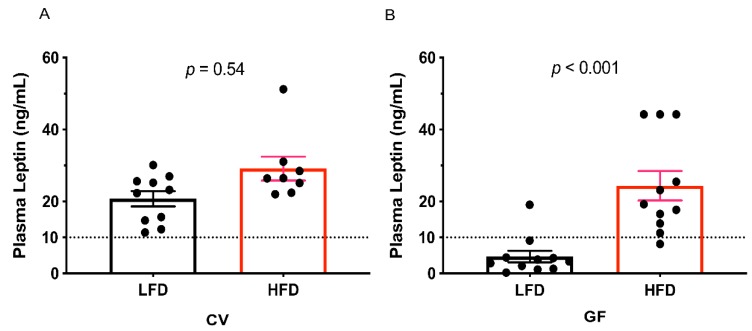
Both the IMB and the HFD increase plasma leptin concentrations in male SW mice. Quantification of plasma leptin concentrations in (**A**) CV (*n* = 10) and (**B**) GF (*n* = 11) mice on the LFD control or the HFD. The values for individual mice are shown as dots, the means as bars, and horizontal lines indicate the standard error of the mean (± SEM). The dotted horizontal lines indicate the cut-off for elevated concentrations (≥10 mg/dL), indicative of hyperleptinemia [28]. Abbreviations: CV conventional; GF, germ-free; HFD, high fat diet; IMB, intestinal microbiota; LFD, low fat diet.

Circulating leptin concentrations are known to increase exponentially with fat mass [55]. Treatment differences in fat pad weights were reflected in plasma leptin concentrations, as we observed high plasma leptin concentrations (≥ 10 mg/dL; as defined for mice [28]) in all CV mice, and all but one GF mouse on the HFD (Figure 2A,B, respectively; Table 3). In the GF mice on the LFD, the fat pad weight was significantly lower than CV mice on either an LFD or HFD (*p* < 0.001, Table 3). Furthermore, for GF mice the HFD significantly increased the fat pad size (*p* < 0.001, Table 3), but this was not observed in the CV Tac:SW mice (Table 3). In all mice we observed a correlation between fat pad size and plasma leptin concentrations (*r* = 0.68, *p* < 0.001, Table 3).

### 3.2. Conventional and Germ-Free Tac:SW Mice Develop Impaired Glucose Regulation on the High Fat Diet

**Figure 3:**
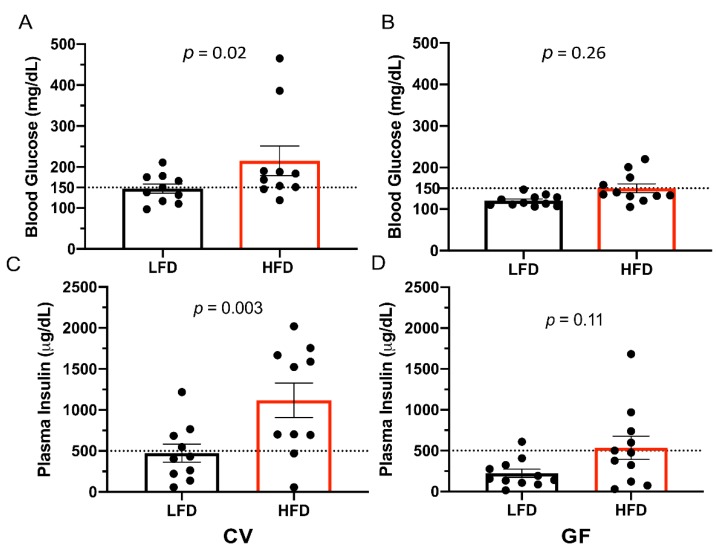
The HFD increases blood glucose and plasma insulin levels in only CV male Tac:SW mice. Quantification of blood glucose concentrations (panels **A**,**B**) and plasma insulin concentrations (panels **C**,**D**) in CV (*n* = 10) and GF (*n* = 11) mice on the LFD control or the HFD. The values for individual mice are shown as dots, the means as bars, and horizontal lines indicate the standard error of the mean (±SEM). The dotted horizontal lines indicate elevated concentrations. Abbreviations: CV, conventional; GF, germ-free; HFD, high fat diet; LFD, low fat diet; Tac:SW, Taconic Swiss Webster.

**Figure 4:**
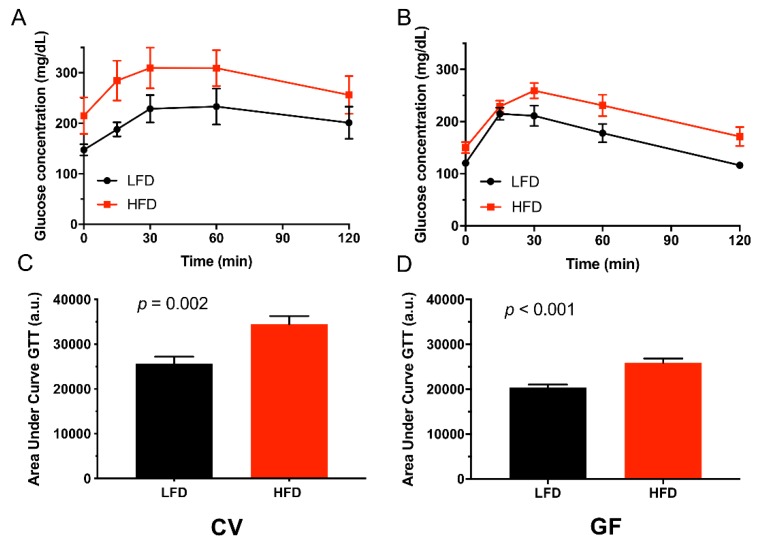
The HFD impairs glucose clearance. Results of the GTT (panels **A,B**) in CV (*n* = 10) and GF (*n* = 11) mice on the LFD control or the HFD. The values for individual mice are shown as dots and horizontal lines indicate the standard error of the mean (± SEM). The calculated area under the curve is shown in panels **C** and **D**, for CV and GF mice, respectively. Statistical analysis via two-sided Student’s t-test, statistical significance was set at *p* ≤ 0.05. Abbreviations: CV, conventional; GF, germ-free; GTT, glucose tolerance test; HFD, high fat diet; LFD, low fat diet.

Following a 6-h daytime fasting period, basal plasma glucose levels for all mice resembled those observed for C57BL/6J mice [56]. The HFD increased fasting glucose and insulin independently of the increase associated with the presence of IMB (Table 4). In all groups (except GF mice on a LFD), some mice reached fasting concentrations of blood glucose ≥ 150 mg/dL indicative of type II diabetes (Table 4; Figure 3A,B) [57]. In all groups (except GF mice on an LFD), we also observed that some mice reached insulin concentrations ≥ 500 µg/dL, with the highest insulin concentrations found in CV mice on the HFD (Figure 3C,D). Furthermore, the GTT showed impaired glucose absorption and insulin sensitivity as defined by glucose concentrations > 150 mg/dL 2-h after glucose injection [58] (Table 4). The HFD impaired glucose clearance in CV and GF mice, as indicated by a statistically significant increase in area under the curve (Figure 4; Table 4). The differences in fasting glucose and insulin concentrations between CV and GF Tac:SW mice are indicative of differences in the development of impaired glucose regulation that exist between the groups. However, the presence of the IMB was not required for GF mice to develop impaired glucose clearance on the HFD. Furthermore, the IMB induced impaired glucose tolerance in CV mice on the LFD.

### 3.3. Conventional and Germ-Free Tac:SW Mice Develop Impaired Lipid Metabolism and Hepatic Lipid Accumulation on the High Fat Diet

**Figure 5:**
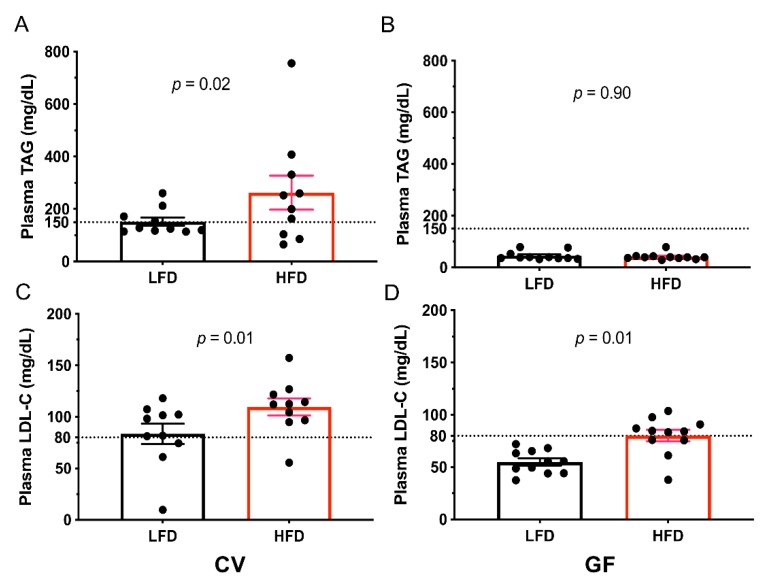
The HFD increases plasma TAG in only CV mice and insulin levels in both CV and GF mice. Quantification of (panels **A**,**B**) plasma triacylglycerol (TAG) and (panels **C**,**D**) LDL-cholesterol (LDL-C) concentrations. The values for individual mice are shown as dots (CV, *n* = 10; GF, *n* = 11), the means as bars, and horizontal lines indicate the standard error of the mean (± SEM). The dotted horizontal lines indicate elevated concentrations. Abbreviations: CV, conventional; GF, germ-free; HFD, high fat diet; LFD, low fat diet; TAG, triacylglycerol.

The presence of the IMB increased plasma lipid concentrations in male Tac:SW mice (Table 5). Because murine cutoff values are not reported, we used human cutoff values for elevated TAG concentrations (>150 mg/dL) [59] and LDL-C concentrations (>130 mg/dL) as reference points [60]. Nearly half of the CV male Tac:SW mice possessed elevated TAG concentrations on the LFD, and one CV mouse on the HFD had elevated LDL-C (Table 5 and Figure 5A,C). Furthermore, plasma TAG, HDL-C, and LDL-C were higher in CV versus GF mice independent of diet (Table 5). In CV mice, the HFD increased TAG, HDL-C, and LDL-C (Table 5 and Figure 5A,C; *p* = 0.02, 0.01, and 0.01, respectively). In GF mice, the HFD significantly elevated LDL-C (Figure 5D; *p* = 0.01), but we did not observe significant changes in TAG and HDL-C (Table 5 and Figure 5B). In summary, on the LFD the IMB induced impaired lipid metabolism in male Tac:SW mice, which was further exacerbated by the HFD, as indicated by a greater concentration of lipids remaining in circulation as opposed to fat pad deposition. In the absence of the IMB, the HFD increased circulating LDL-C but not TAG concentrations (Table 5); however, we observed that TAG was deposited into the fat pad instead (Table 3). 

The results for hepatic free and acylated (AC) carnitine are shown in Table 6. The presence of the IMB increased the relative abundance of short-chain acylcarnitines (*p* = 0.002), which are partly derived from short-chain fatty acids (SCFAs) excreted by the IMB [61]. Furthermore, presence of the IMB modified the effect of the HFD on medium- (*p* = 0.004) and long-chain acylcarnitines (*p* < 0.006). In CV mice, the HFD increased the relative abundance of medium-chain acylcarnitines by 144% (*p* < 0.001) and long-chain acylcarnitines by 358% (*p* = 0.001). In contrast, no consistent effect was observed on medium- (*p* = 0.99) and long-chain acylcarnitines (*p* = 0.58) in GF mice, similar to what we observed for plasma TAG (Table 6).

The results for hepatic lipid levels are shown in Table 7 and for individual lipid species in Appendix A Table A1. Presence of the IMB increased liver weight by 86% ((average of GF mice compared to average of CV mice) *p* < 0.001; Table 7). Within the liver lipid extract, the HFD increased relative abundance of TAG by 41% in both CV and GF mice. Specifically, TAG with a higher degree of unsaturation were increased (Appendix A Table A1); in contrast the relative abundance of TAG with shorter chain lengths (TAG < C52) were decreased (Appendix A Table A1). In addition to TAG, the HFD increased the relative abundance of sphingolipids, including ceramides, by 35% in both CV and GF mice (*p* < 0.001). In GF mice, the HFD also increased the relative abundance of free fatty acids (FFA) by two-fold (Table 7). Presence of the IMB did not alter the levels of TAG but decreased the relative abundance of sphingolipids, glycerophospholipids (GPL), and FFA (Table 7). Thus, presence of the IMB was not required for the HFD to promote TAG and ceramide accumulation in the liver. Furthermore, presence of an IMB decreased hepatic glycerophospholipid, sphingolipid, and FFA levels, but did not alter hepatic TAG levels.

## 4. Discussion

It is well established that males predominantly develop symptoms associated with DIO, MetS, and T2DM [39]. In one breeding colony of Swiss Webster mice at the Massachusetts Institute of Technology Division of Comparative Medicine, researchers observed obesity in some female mice, but no diabetic females were identified. In the same brood, the majority of males exhibited severe symptoms of DIO and T2DM [39]. Furthermore, in other mouse strains females are protected from the symptoms associated with DIO [48,49,50]. Previous research showed that only ovariectomized female mice resemble male mice in their susceptibility to weight gain and DIO [49]. As the majority of prior studies comparing CV and GF mice were done with males, we focused on male CV and GF Swiss Webster mice in this study. The effect of an HFD on body weight and metabolism of glucose and lipids is well established in male CV mice [62,63]; however, less is known about the effect of an HFD in GF mice. Studies comparing male CV and GF C57BL/6J, C57BL/6N, and C3H mice suggest diet composition has a major impact on weight gain in the GF state [10,31,32,34]. However, the mouse strain could also affect the outcome, as strain-specific differences in CV mice lead to varying degrees of DIO [64]. Therefore, the strain’s genetic background must be taken into account in DIO studies. GF Tac:SW mice are frequently used in fecal transplant studies and are proposed for use in a simplified human IMB model to study diet–host–microbiota interactions in relation to metabolic diseases [40]; however, the role that the IMB play in the development of obesity and MetS in Tac:SW mice is unknown. Our findings are the first to demonstrate that outbred GF male Tac:SW mice develop many characteristics of DIO and MetS.

The paramount phenotype associated with DIO is weight gain. We observed that both CV and GF male Tac:SW mice on the HFD more than doubled their average daily weight gain compared with mice receiving the LFD, over a 10-week feeding study. The weight gain observed in the CV male Tac:SW mice stood in contrast to a previous study, which indicated outbred ND4 Swiss Webster mice (Harlan Industries, Placentia, CA, USA) are protected from DIO, even after consuming an HFD (60% kcal from fat) for 33 weeks [65]. However, a subsequent study showed CV Swiss Webster mice (University of São Paulo, Brazil) are susceptible to weight gain on an HFD [46]. In our study, the increased rate of weight gain led to a significant increase in body weight for mice on the HFD compared to the LFD control, independent of IMB status. Therefore, absence of the IMB did not protect male Tac:SW mice from an increase in body weight. It is worth noting that GF Tac:SW mice on the LFD diet did not gain significant weight until week 10, and that the average weight remained level or dropped over the course of the 10-week feeding study. We attribute this effect to fluctuations in cecal volume, as the cecum of GF mice is dramatically enlarged compared to CV mice, and its volume can change dramatically with diet [66,67]. The observed phenotype in GF male Tac:SW mice is similar to GF male C3H mice on an HFD [32]. However, where we used lard, a saturated, cholesterol-containing lipid, as the fat source in the HFD (Table 2), Fleissner et al. used coconut oil, which is rich in saturated fats but lacks cholesterol as the fat source in the C3H study [32]. Therefore, the results of the C3H study mirror those observed by Kübeck et al. with C57BL/6N mice, where GF mice gained weight on a cholesterol-free palm oil-based HFD [34]. Our GF Tac:SW mice gained weight on the low-sucrose lard-based HFD, whereas GF C57BL/6N mice did not [34]. On the other hand, several studies found that adult GF male C57BL/6J mice do not develop obesity on an HFD [10,29,31]. In contrast, Caesar et al. found that GF male C57BL/6J mice gained weight when fed a lard based HFD [33]. Taken together, these results suggest that both diet and strain differences play a role in the development of DIO.

In rodents, ad libitum access to HFD increases the daily caloric intake, and leads to weight gain [68]. While an HFD is capable of invoking mechanisms that limit total daily caloric intake, C57BL/6 mice over-consume an HFD when provided ad libitum [13]. We found that male Tac:SW mice developed obesity on the HFD; however, unlike male C57BL/6 mice, CV male Tac:SW mice reduced their feed intake on an HFD, suggesting a strain difference. On the other hand, GF male Tac:SW mice did not reduce their feed intake when fed the HFD, a difference that could involve the IMB. Furthermore, we observed higher feed consumption in CV versus GF mice on the LFD, which was noted previously in C57BL/6J mice [31]. Our male GF Tac:SW mice on the HFD were similar to GF male C3H mice on an HFD, as they possessed larger fat pads than their CV counterparts [32]. We observed that the HFD invoked greater feed conversion in both CV and GF mice. A plausible explanation for the increase in feed conversion is a microbiota-independent increase in lipid absorption with an HFD, as lipids are well absorbed in the ileum and jejunum [69,70]. There was an additive effect of IMB on feed conversion, with a greater increase in feed conversion when comparing mice on the HFD with those on the LFD. The IMB utilize carbohydrates and deaminated amino acids to excrete SCFAs, and also aid digestion of complex dietary polysaccharides into sugars digestible by the host [61,71]. The intestine absorbs these SCFAs and the liver utilizes them to synthesize glucose and fatty acids [61], which could explain the increase in feed conversion caused by the IMB. Finally, we found that GF male Tac:SW mice weighed less and possessed smaller livers than their age-matched CV counterparts, pointing towards a role of the IMB in growth and nutrient utilization, described previously [72]. 

Leptin plays an important role in DIO, as circulating leptin levels increase exponentially with an increase in fat mass [55]. Therefore, circulating leptin levels reflect the amount of fat stored but also indicate an energy imbalance [55]. Leptin is produced predominantly by adipose tissue, and leptin induces satiety through leptin receptors in the hypothalamus [73]. Obesity elevates circulating leptin concentrations (i.e., hyperleptinemia), which in turn causes leptin resistance of the hypothalamus and promotes further weight gain [74]. Elevated circulating leptin concentrations were previously observed in male CV Swiss Webster mice (University of São Paulo, Brazil) on a high sucrose lard-based HFD [46]. In our study, all groups with marked weight gain possessed elevated plasma leptin concentrations (>10 ng/mL), which may signify hyperleptinemia [28]. In contrast, adult male GF or CV C57BL/6J mice did not develop elevated leptin concentrations on an 8-week high-fat, high-sucrose, calorie-dense diet [10]. It is interesting to note that elevated leptin concentrations were found in all CV male Tac:SW mice on the LFD. Furthermore, these same mice also gained weight on the LFD, and possessed similarly sized fat pads to their counterparts on an HFD. GF male Tac:SW mice on an LFD did not gain weight until the last week of the study. It is possible to attribute this phenotype to the reduced energy intake and enhanced lipid secretion found in GF mice [31]. Interestingly, our high corn starch LFD can cause obesity in CV male Tac:SW mice, which stands in contrast to a previous study that used diets high in sucrose to show that the adiposity of C57BL/6J mice and four other inbred strains was primarily due to the fat content of the diet [75].

Obesity remains the leading risk factor in the development of T2DM [76]. In this study, the HFD induced features of T2DM in some of the mice, illustrated by elevated fasting glucose and insulin concentrations, and impaired glucose tolerance/insulin resistance as determined by a GTT. This contrasts with previous studies that observed a significant increase in fasting levels of insulin, but not glucose, in male CV Swiss Webster mice on a high-sucrose lard-based HFD and C57BL/6J on an HFD (45% kcal from fat) [29,46]. The fasting levels of insulin we observed were highest in CV mice on the HFD, but these were also high in half of the GF mice on the HFD and half of the CV mice on the LFD. The inability of our mice to maintain their circulating concentrations of glucose and insulin within a healthy range is a critical step in the pathway from obesity to chronic diseases; however, impaired glucose metabolism can develop in the absence of obesity [77]. A HFD diet can promote elevated glucose concentrations in circulation because the glycerol unit of TAG can be converted to glucose [78]. Glucose is needed, because the oxaloacetate needed for β-oxidation is formed by the carboxylation of pyruvate, which is a product of glycolysis [79]. Therefore, an easily digestible carbohydrate-rich diet can both promote β-oxidation and elevate circulating glucose concentrations, even in the absence of excessive weight gain in GF mice [10]. The microbiota provide additional gluconeogenesis precursors from endogenously undigestible fiber as well as from TAG-derived glycerol and can further increase glucose concentrations, as reported previously [10]. Our findings with GF Tac:SW mice diverge from those with GF C57BL6J and C3H mice, in that we observed elevated insulin concentrations with the HFD, which was not the case in the other two studies [10,32]. Plasma insulin concentrations were not provided in the C57BL/6N study [34]. 

Another important step on the pathway from obesity to chronic disease is dyslipidemia, which is defined in humans by elevated TAG and LDL-C concentrations, and low HDL-C concentrations in circulation [80]. Elevated TAG and LDL-C signify excess lipids in circulation that can form plaques, but also signify an inability of tissue absorption or cholesterol excretion [81]. However, mice differ from humans as the higher HDL-C concentrations in mice partly protect non-genetically modified mice from elevated, circulating TAG and LDL-C concentrations by removing excess cholesterol [81]. In our CV Tac:SW mice, unlike CV ND4 Swiss Webster mice (Harlan Industries, Placentia, CA, USA), the HFD diet increased circulating concentrations of TAG, HDL-C, and LDL-C, suggesting that they could not clear excess dietary lipids [65]. In contrast, on the HFD, GF mice did not show increased circulating lipid concentrations except for LDL-C, suggesting a potential role of the IMB in the development of hypertriglyceridemia in male Tac:SW mice. Insufficient lipid absorption in the gut of GF mice due to changes in the bile acid composition of GF mice could explain this observation [31,82]. An important pathway that removes excess TAG and cholesterol from circulation is tissue absorption, but excess intracellular TAG accumulation in tissue can impair tissue function [83]. For example, excess TAG accumulation in pancreatic tissue can impair insulin secretion, demonstrating a link between impaired glucose and TAG metabolism [83]. In our study, increased hepatic TAG accumulation occurred independent of the IMB. The accumulated TAG reflected the fatty acid composition of the diet, which predominantly contained unsaturated fatty acids (62%).

Fatty acid oxidation in liver and muscle are essential ways to remove TAG from circulation. Carnitines play an important role in hepatic and muscle lipid oxidation, as they transport long-chain FA as acylcarnitines from the cytosol into the mitochondria for β-oxidation [84]. When β-oxidation exceeds the capacity of the TCA cycle, acylcarnitines of various carbon-chain length accumulate in the circulation, which can adversely affect insulin sensitivity [85,86]. Alternatively, SCFAs can be converted into ketone bodies and excreted in the urine [87]. In CV Tac:SW mice, hepatic accumulation of medium- and long-chain acylcarnitines occurred in response to the HFD, an indication that hepatic β-oxidation could not keep up with the increased supply of fatty acids from the HFD. In contrast, no changes in hepatic acylcarnitine abundance was observed in GF Tac:SW mice. Considering the observed increase in fat pad weight in the GF mice, we hypothesize that the increased supply of fatty acids from the HFD was not oxidized in the liver but rather transferred as TAG to the adipose tissue. A similar observation was made for male CV and GF C3H mice on a low-sucrose lard-based HFD [32]. In contrast, this was not observed with male GF C57BL/6J mice on a Western diet [10,11]. Furthermore, in our study the LFD caused hepatic lipid accumulation even in GF Tac:SW mice, with both CV and GF mice possessing similar hepatic TAG concentrations, which confirms that TAG uptake in the intestine is not impaired by the lack of the IMB [88]. Diet composition matters as well, we would expect lower hepatic TAG concentrations in GF Tac:SW mice fed a normal chow diet than what we observed using the LFD [88]. It is interesting to note that in our study the IMB increased the levels of the most abundant acylcarnitine, acetyl-L-carnitine (AC2:0-carnitine), and its abundance was not affected by the HFD. Acetate production by the IMB mostly escapes first pass metabolism in the liver, but it can aid in lipogenesis by providing acetyl units and could explain why male CV Tac:SW mice on the LFD gained weight, whereas GF mice on the same diet did not [89]. 

Obesity, T2DM, and NAFLD are characterized by the presence of chronic, low-grade inflammation. This chronic low-grade inflammation promotes the occurrence of metabolic anomalies, such as insulin resistance and dyslipidemia [90]. One of the drivers of this inflammation are sphingolipid metabolites, such as ceramides, which are important for the signaling of lipid-induced inflammatory pathways [91]. We noted a hepatic increase in sphingolipids (i.e., sphingomyelin and ceramides) in response to the HFD. Ceramides have been linked to hepatic steatosis, but conflicting results in human studies do not allow us to draw an association between an increase in hepatic ceramide levels and the development of hepatic steatosis [92]. Elevated plasma concentrations of inflammatory markers, including IL1β and IL6, in response to the HFD were not observed in our study (data not shown), indicating that these male Tac:SW mice had not yet developed a pro-inflammatory phenotype [93]. In conclusion, we propose that in male Tac:SW mice the IMB is a risk factor, but not a requirement in developing symptoms associated with DIO. GF male Tac:SW mice develop many characteristics of DIO and MetS, which supports their use in studying diet–host–microbiota interactions in relation to these conditions.

## Figures and Tables

**Table 1:** Summary of diet-induced obesity (DIO) in germ-free (GF) mice.

**Author/Year**	Bäckhed et al. [10]	Fleissner et al. [33]	Rabot et al. [31]	Ding et al. [29]	Caesar et al. [34]	Kübeck et al. [35]
**Mouse Strain**	C57BL/6J	C3H	C57BL/6J	C57BL/6J	C57BL/6J	C57BL/6N
**Diet Type**						
**Fat (%)**	40.6% 50% lard + 50% hydrogen-ated vegetable shortening	Diet 1, 43% 90% coconut + 5% thistle + 5% linseed oil Diet 2, 40.6% 50% lard + 50% hydrogen-ated vegetable shortening	60% 94% lard + 6% soybean oil	45% 88% lard, 12% soybean oil	45% Diet 1: 88% lard + 12% soybean oil Diet 2: 88% fish oil + 12% soybean oil	48% Diet 1: 80% lard + 20% soybean oil Diet 2: 80% palm oil + 20% soybean oil
**Carbohy-drate% Sucrose (g/kg)**	40.7 (183)	41 (Diet 1: 50; Diet 2: 183)	20 (73)	35 (0)	35 (173)	34 (50)
**Protein%**	18.7	16	20	20	20	18
**Suscepti-ble to DIO?**	No	Diet 1: Yes Diet 2: No	No	No	Diet 1: Yes Diet 2: No	Diet 1: No Diet 2: Yes
**Findings**	GF mice protected from DIO by increased AMPK activity and FA oxidation in peripheral tissues and increased intestinal *Fiaf* expression.	Diet 1: gained significant weight. Diet 2: no weight gain as observed by Bäckhed et al.(10). Composition of lipid and CHO affects weight gain. No role for intestinal *Fiaf* or SCFA.	GF mice consume fewer calories and excrete more fecal lipids than CV mice, gain less weight on HFD.	HFD and gut bacteria interact to promote IFM that precedes develop-ment of obesity, adiposity and insulin resistance in CV mice.	Lard-based HFD activates TLR signaling, induces CCL2, subsequent macrophage recruitment to WAT and IFM. GF mice only partly protected from lard-induced WAT IFM indicating microbiota-independent mechanisms contribute to IFM.	Cholesterol affects the crosstalk between IMB and host metabolism. Diet composition matters, GF animals on lard-based HFD saw reduced intestinal fat absorption

Abbreviations: AMPK, AMP-activated protein kinase; CCL2, C-C motif chemokine ligand 2; CHO, carbohydrate; CV, conventional; FA, fatty acid; *Fiaf*, fasting-induced adipose factor; HFD, high fat diet; IFM, inflammation; IMB, intestinal microbiota; SCFA, short chain fatty acid; TLR, toll-like receptor; WAT, white adipose tissue.

**Table 2:** Experimental diet composition.

	Low-Fat Diet		High-Fat Diet	
Ingredient	g/kg	kcal/kg	g/kg	kcal/kg
Lard	19.0	171.0	316.6	2849.4
Corn oil	23.7	213.3	32.3	290.7
Cornstarch	411.9	1482.8	0.0	0.0
Dyetrose ^1^	161.6	614.1	161.6	614.1
Sucrose	88.9	355.6	88.9	355.6
Cellulose	47.4	0	54.6	0
Casein	189.6	678.8	258.5	925.4
Mineral mix #210088 ^1^	10.0	16.0	12.9	20.6
Vitamin mix #300050 ^1^	10.0	39.2	12.9	50.6
Dicalcium carbonate	12.3	0	16.8	0
Potassium citrate	15.6	0	21.3	0
Choline bitartrate	2.0	0	2.6	0
Total energy, kcal		3571		5106
Total fat, g	43.0		349.0	
14:0 Myristic acid	0.3		7.8	
16:0 Palmitic acid	7.7		84.3	
16:1 Palmitoleic acid	0.7		10.8	
18:0 Stearic acid	3.7		42.9	
18:1 Oleic acid	14.5		147.0	
18:2 Linoleic acid	15.9		51.9	
18:3 Linoleic acid	2.3		7.3	
Total carbohydrate, g	673		263	
Total fiber, g	47.4		64.6	
Total protein, g	192.0		262.0	
Total cholesterol, g	0.014		0.28	

^1^ Dyetrose and mineral and vitamin mixes are proprietary products of Dyets, Inc.

**Table 3:** Effect of the low- and high-fat diets on body and tissue weight, feed consumption, and feed conversion in conventional and germ-free male Tac:SW mice.

	Conventional		Germ-Free			*p*-Values		
Parameter	LFD	HFD	LFD	HFD	SEM	IMB	HFD	IMB × HFD
Body Weight	*n* = 10	*n* = 10	*n* = 11	*n* = 11				
Starting, g	41.9	44.1	39.1	38.0	1.1	<0.001 ^c^	0.64	0.13
Final, g	48.0 ^b^	56.0 ^a^	41.5 ^b^	46.3 ^a^	1.7	<0.001 ^c^	<0.001 ^c^	0.34
DWG, mg/d	88 ^b^	170 ^a^	36 ^b^	118 ^a^	16	0.002 ^c^	<0.001 ^c^	0.99
Fat pad, g	1.86	1.77	0.77 ^b^	2.77 ^a^	0.28	0.86	<0.001 ^c^	<0.001 ^e^
Fat pad, %BW	3.81	3.13	1.87 ^b^	5.85 ^a^	0.46	0.35	<0.001 ^c^	<0.001 ^e^
Feed								
Consumption g/d	4.19 ^a^	3.44 ^b^	3.82	3.68	0.14	0.65	0.003 ^d^	0.03 ^e^
Conversion, %	2.09 ^b^	4.97 ^a^	0.94 ^b^	3.22 ^a^	0.43	0.001 ^c^	<0.001 ^c^	0.48
Plasma								
Leptin, ng/mL	20.75	23.40	4.69 ^b^	24.37 ^a^	2.75	0.01 ^c^	<0.001 ^c^	<0.001 ^e^
High Leptin, *n* =	10	10	1 ^b^	10 ^a^		0.002 ^c^	0.004 ^c^	0.004 ^e^

High leptin was defined as plasma leptin concentrations ≥ 10 mg/dL [28]. Superscripts “a” (larger numerical value) and “b” (smaller numerical value) denote statistically significant (*p* ≤ 0.05) diet-induced differences within CV and within GF mice, respectively. Superscript “c” indicates a statistically significant increase caused by the microbiota, or the HFD. Superscript “d” indicates a statistically significant decrease caused by the intestinal microbiota or the HFD, and superscript “e” indicates a statistically significant interaction. The SEM is a pooled standard error of the mean. Analysis was performed using the method of least squares to fit general linear models in SAS. The *p* values shown are for the main effects of IMB, main effect of HFD, and their interaction/effect modification (IMB × HFD). “n =” indicates the number of mice with this characteristic per column. Abbreviations: Tac:SW, Taconic Swiss Webster; DWG, daily weight gain; BW, body weight; CV, conventional; GF, germ-free; IMB, intestinal microbiota; HFD, high fat diet.

**Table 4:** Effect of the low- and high-fat diets on glucose metabolism in conventional and germ-free male Tac:SW mice.

	Conventional		Germ-Free			*p*-Values		
	LFD	HFD	LFD	HFD	SEM	IMB	HFD	IMB × HFD
Fasting	*n* = 10	*n* = 10	*n* = 11	*n* = 11				
Glucose, mg/dL	147 ^a^	215 ^a^	120	150	19	0.02 ^c^	0.01 ^c^	0.32
Insulin, µg/dL	473 ^b^	1118 ^a^	223	536	142	0.005 ^c^	0.001 ^c^	0.24
High glucose, *n* =	5	8	0	4		0.004 ^c^	0.06	1
High insulin, *n* =	4	8	1	5		0.06	0.03 ^c^	1
Glucose tolerance test						0.03 ^c^	0.01 ^c^	0.42
Baseline	147 ^a^	215 ^a^	120	150	25	0.02 ^c^	0.01 ^c^	0.32
15 min	188 ^a^	285 ^a^	215	229	25	0.51	0.01 ^c^	0.07
30 min	229 ^a^	310 ^a^	211	259	25	0.21	0.02 ^c^	0.54
60 min	233	309	178	231	25	0.02 ^c^	0.03 ^c^	0.68
120 min	201	256	116	171	25	0.002 ^c^	0.04 ^c^	0.99
Impaired GTT *n* =	6	8	0 ^b^	5 ^a^		0.005 ^c^	0.06	0.54

High glucose was defined as fasting plasma glucose concentrations ≥ 150 mg/dL [57]. High insulin was defined as fasting insulin concentrations ≥ 500 µg/dL. Impaired glucose tolerance was defined as plasma fasting glucose concentrations ≥ 150 mg/dL 120 min after glucose injection [58]. Superscripts “a” (larger numerical value) and “b” (smaller numerical value) denote statistically significant (*p* ≤ 0.05) diet-induced differences within CV and within GF mice, respectively. Superscript “c” indicates a statistically significant increase caused by the microbiota, or the HFD. The SEM is a pooled standard error of the mean. Analysis was performed using the method of least squares to fit general linear models in SAS. The *p* values shown are for the main effects of microbiota (IMB), main effect of diet (HFD), and their interaction/effect modification (IMB × HFD). “n =” indicates the number of mice with this characteristic per column. Abbreviations: CV, conventional; GF, germ-free; GTT, glucose tolerance test; HFD, high fat diet; IMB, intestinal microbiota; LFD, low fat diet; Tac:SW, Taconic Swiss Webster.

**Table 5:** Effect of the low- and high-fat diets on plasma lipid concentrations in conventional and germ-free male Tac:SW mice.

	Conventional		Germ-Free			*p*-Values		
Parameter	LFD	HFD	LFD	HFD	SEM	IMB	HFD	IMB × HFD
Plasma (mg/dL)	*n* = 10	*n* = 10	*n* = 11	*n* = 11				
Triacylglycerol	152 ^b^	237 ^a^	46	42	24	<0.001 ^c^	0.09	0.06
HDL-C	74 ^b^	119 ^a^	53	62	12	0.002 ^c^	0.03 ^c^	0.14
LDL-C	92 ^b^	110 ^a^	55 ^b^	80 ^a^	6	<0.001 ^c^	<0.001 ^c^	0.49
High-TAG, *n* =	4	7	0	0		<0.001 ^c^	0.48	0.48
High LDL-C, *n* =	4	7	0	1		<0.001 ^c^	0.18	0.73
LDL-C ≥ 80, *n* =	8	9	0 ^b^	7 ^a^		<0.001 ^c^	0.03 ^c^	0.06

High triacylglycerol (High-TAG) was defined as fasting TAG concentrations ≥150 mg/dL [59]. High low-density lipoprotein-cholesterol (High LDL-C) was defined as fasting LDL-C concentrations ≥80 mg/dL. Superscripts “a” (larger numerical value) and “b” (smaller numerical value) denote statistically significant (*p* ≤ 0.05) diet-induced differences within CV and within GF mice, respectively. Superscript “c” indicates a statistically significant increase caused by the microbiota, or the HFD. The SEM is a pooled standard error of the mean. Analysis was performed using the method of least squares to fit general linear models in SAS. The *p* values shown are for the main effects of IMB, main effect of HFD, and their interaction/effect modification (IMB × HFD). “n =” indicates the number of mice with this characteristic per column. Abbreviations: CV, conventional; GF, germ-free; HFD, high fat diet; IMB, intestinal microbiota.

**Table 6:** Effect of the low- and high-fat diets on hepatic carnitine levels in conventional and germ-free male Tac:SW mice.

	Conventional		Germ-Free			*p*-Values		
Peak Area × 10^3^	LFD	HFD	LFD	HFD	SEM	IMB	HFD	IMB × HFD
Carnitines	*n* = 10	*n* = 10	*n* = 11	*n* = 11				
Free	71,457	67,040	69,794	69,217	3482	0.94	0.47	0.58
acylated (AC)								
**Total**	105,362	118,749	86,036	80,669	10,614	0.009 ^c^	0.70	0.37
Short-Chain	99,201	91,191	70,044	67,839	7905	0.002 ^c^	0.51	0.71
AC 2:0	89,244	76,008	58,864	55,346	7182	<0.001^c^	0.24	0.49
AC 4:1-OH	9034	14,012	10,509	11,992	2631	0.92	0.22	0.50
AC 5:0	923	1171	671	501	194	0.02 ^c^	0.84	0.28
Medium-Chain	296 ^b^	722 ^a^	307	308	72	0.007 ^d^	0.004 ^c^	0.004 ^e^
AC 8:0	127	149	128 ^b^	639^a^	20	0.04 ^d^	0.28	0.03 ^e^
AC 10:0	119 ^b^	214 ^a^	118	119	279	0.08	0.08	0.09
AC 14:2	49 ^b^	358 ^a^	61	125	37	0.004 ^d^	<0.001^c^	0.002 ^e^
Long-Chain	5865 ^b^	26,836^a^	15,685	12,522	4218	0.59	0.04 ^c^	0.006 ^e^
AC 16:0	995 ^b^	4458 ^a^	2562	2756	770	0.93	0.02 ^c^	0.04 ^e^
AC 16:1	913 ^b^	2138 ^a^	2072 ^a^	726 ^b^	380	0.74	0.87	0.001 ^e^
AC 18:0-OH	52 ^b^	178 ^a^	108 ^b^	181 ^a^	24	0.22	<0.001^c^	0.26
AC 18:1-OH	159 ^b^	591 ^a^	480	382	87	0.52	0.06	0.004 ^e^
AC 18:1	2542 ^b^	12,621^a^	7591	4273	2284	0.46	0.14	0.005 ^e^
AC 18:2	591 ^b^	3816 ^a^	965	2291	559	0.63	<0.001^c^	0.09
AC 18:3	59 ^b^	291 ^a^	80	163	47	0.25	0.002 ^c^	0.12
AC 20:1	295 ^b^	1475 ^a^	1170 ^a^	602 ^b^	192	0.99	0.11	<0.001 ^e^
AC 20:2	136 ^b^	833 ^a^	297	365	87	0.08	<0.001^c^	0.0007 ^e^
AC 20:4	122 ^b^	435 ^a^	359 ^b^	782 ^a^	64	<0.001^d^	<0.001^c^	0.38

All values are expressed in peak area x 10^3^. Superscripts “a” (larger numerical value) and “b” (smaller numerical value) denote statistically significant (*p* ≤ 0.05) diet-induced differences within CV and within GF mice, respectively. Superscript “c” indicates a statistically significant increase caused by the microbiota, or the HFD. Superscript “d” indicates a statistically significant decrease caused by the intestinal microbiota or the HFD, and superscript “e” indicates a statistically significant interaction. The SEM is a pooled standard error of the mean. Statistical analysis was performed using the method of least squares to fit general linear models in SAS. The *p* values shown are for the main effects of microbiota (IMB), main effect of diet (HFD), and their interaction/effect modification (IMB × HFD). Abbreviations: CV, conventional; GF, germ-free; HFD, high fat diet; IMB, intestinal microbiota Tac:SW, Taconic Swiss Webster.

**Table 7:** Effect of the low- and high-fat diets on hepatic lipid abundance in conventional and germ-free male Tac:SW mice.

		Conventional		Germ-Free			*p*-Values		
Parameter Species		LFD	HFD	LFD	HFD	SEM	IMB	HFD	IMB × HFD
Liver		*n* = 10	*n* = 10	*n* = 11	*n* = 11				
Liver, g		2.59	2.88	1.46	1.48	0.27	<0.001 ^c^	0.52	0.58
Liver, %BW		5.35	5.16	3.55	3.24	0.44	<0.001 ^c^	0.53	0.88
Liver lipid extract (peak area × 10^3^)								
Total lipids	125	36,892 ^b^	49,017 ^a^	44,975 ^b^	54,577 ^a^	3025	0.03 ^d^	<0.001 ^c^	0.67
Acylglycerols	45	18,455 ^b^	27,413 ^a^	19,936 ^b^	26,728 ^a^	1413	0.77	<0.001 ^c^	0.44
TAG	34	18,156 ^b^	27,054 ^a^	19,575 ^b^	26,269 ^a^	1393	0.82	<0.001 ^c^	0.42
DAG	9	258	314	314	409	51	0.14	0.14	0.70
MAG	2	40	45	48	50	18	<0.001 ^d^	0.05 ^c^	0.57
Glycero PL	65	14,258	16,448	19,797	22,027	2211	0.01 ^d^	0.31	0.99
PA	6	155	181	216 ^b^	332 ^a^	237	<0.001 ^d^	0.004 ^c^	0.06
Lyso-PE	6	165	170	209	239	35	0.10	0.61	0.72
PE	15	7888	9472	11,315	13,161	1329	0.009 ^d^	0.19	0.92
PC	16	3418	3711	4100	4489	454	0.13	0.47	0.92
PI	10	1484	1633	2357 ^a^	1634 ^b^	2272	0.06	0.20	0.06
PS	7	838	1087	1310 ^b^	1928 ^a^	147	<0.001 ^d^	0.004 ^c^	0.20
PG	5	310 ^a^	194 ^b^	290	243	282	0.60	0.006 ^c^	0.23
Sphingolipids	9	2105 ^b^	3412 ^a^	3560 ^b^	4254 ^a^	184	<0.001 ^d^	<0.001 ^c^	0.10
Ceramides	5	315 ^b^	491 ^a^	513 ^b^	669 ^a^	33	<0.001 ^d^	<0.001 ^c^	0.77
Sphingomyelin	3	1790 ^b^	2920 ^a^	3047 ^b^	3585 ^a^	171	<0.001 ^d^	<0.001 ^c^	0.08
FFA	6	44	52	83 ^b^	166 ^a^	28	0.008 ^d^	0.10	0.18

All values are sums of individual lipid species and expressed in peak area × 10^3^. Only classes and groups >1 lipid species are shown. Superscripts “a” (larger numerical value) and “b” (smaller numerical value) denote statistically significant (*p* ≤ 0.05) diet-induced differences within CV and within GF mice, respectively. Superscript “c” indicates a statistically significant increase caused by the microbiota, or the HFD. Superscript “d” indicates a statistically significant decrease caused by the intestinal microbiota or the HFD. The SEM is a pooled standard error of the mean. Analysis was performed using the method of least squares to fit general linear models in SAS. The *p* values shown are for the main effects of IMB, main effect of HFD, and their interaction/effect modification (IMB × HFD). Abbreviations: CV, conventional, DAG, diacylglycerol; FFA, free fatty acid; GF, germ-free; Glycero PL, glycerophospholipids; HFD, high fat diet; IMB, intestinal microbiota; LFD, low fat diet; MAG, monoacylglycerol; PA, phosphatidic acid; PE, phosphatidylethanolamine; PC, phosphatidylcholine; PI phosphatidylinositol; PS, phosphatidylserine; PG, phosphatidylglycerol; Tac:SW, Taconic Swiss Webster; TAG: triacylglycerol.

## Abbreviations

AC	acylated
BW	body weight
CV	conventional
DAG	diacylglycerol
DWG	daily weight gain
FFA	free fatty acid
GF	germ-free
glycero PL	glycerophospholipids
GTT	glucose tolerance test
HDL-C	high density lipoprotein cholesterol
HFD	high-fat diet
IFM	inflammation
IMB	intestinal microbiota
LDL-C	low density lipoprotein cholesterol
LFD	low-fat diet
MetS	metabolic syndrome
PA	phosphatidic acid
PC	phosphatidylcholine
PE	phosphatidylethanolamine
PG	phosphatidylglycerol
PI	phosphatidylinositol
PS	phosphatidylserine
Tac:SW	Swiss Webster (Taconic)
TAG	triacylglycerol.

## Appendix A

**Table A1:** Quantification of the total amount of lipids found per lipid identified by mass spectrometry, as calculated by peak area intensity from the UPLC chromatogram.

				Conventional		Germ-Free		*p*-value		
Lipid Nr.	Lipid Group	Chain Length	Double Bond	Control	HFD	Control	HFD	IMB	Diet	IMB x Diet
1	Ceramide	16	0	43	48	82	71	<0.001	0.6	0.18
2	Ceramide	20	0	24	53	36	80	<0.001	<0.001	0.1
3	Ceramide	22	0	134	257	140	332	0.04	<0.001	0.07
4	Ceramide	24	0	101	120	220	168	<0.001	0.21	0.009
5	Ceramide	24	1	13	14	34	19	<0.001	0.001	0.001
6	SM	16	0	142	186	256	246	<0.001	0.17	0.03
7	SM	18	0	104	289	214	522	<0.001	<0.001	0.09
8	SM	28	0	268	775	272	603	0.11	<0.001	0.1
9	Cholesterol	.	.	1995	1651	1533	1304	0.03	0.11	0.74
10	FFA	16	0	6	7	11	16	0.001	0.15	0.24
11	FFA	18	0	8	10	9	11	0.31	0.03	0.99
12	FFA	18	1	6	4	26	23	<0.001	0.55	0.93
13	FFA	18	2	3	5	8	30	0.003	0.02	0.03
14	FFA	20	4	14	18	20	48	0.13	0.17	0.28
15	FFA	22	6	5	9	10	38	0.007	0.01	0.04
16	MAG	18	0	29	34	38	40	<0.001	0.06	0.49
17	MAG	18	2	11	11	10	11	0.01	0.42	0.53
18	DAG	34	1	72	47	53	43	0.15	0.02	0.3
19	DAG	34	2	65	95	63	111	0.63	0.007	0.52
20	DAG	34	3	10	10	12	12	0.34	0.9	0.73
21	DAG	36	2	30	29	98	46	0.001	0.04	0.04
22	DAG	36	3	30	59	53	124	0.008	0.003	0.18
23	DAG	36	4	19	19	13	17	0.01	0.23	0.14
24	DAG	38	6	26	42	14	27	0.004	0.002	0.69
25	DAG	40	6	3	5	2	4	0.15	<0.001	0.54
26	DAG	40	7	4	8	6	24	0.002	<0.001	0.02
27	TAG	46	2	30	14	63	20	0.003	<0.001	0.04
28	TAG	48	0	29	23	24	24	0.24	0.09	0.1
29	TAG	48	1	273	51	222	37	0.07	<0.001	0.28
30	TAG	48	2	393	80	443	59	0.7	<0.001	0.36
31	TAG	50	1	884	496	581	430	0.003	<0.001	0.05
32	TAG	50	2	2161	716	1954	605	0.09	<0.001	0.6
33	TAG	50	3	1307	589	1261	354	0.11	<0.001	0.27
34	TAG	50	4	251	278	319	206	0.97	0.23	0.05
35	TAG	51	2	155	106	121	65	<0.001	<0.001	0.65
36	TAG	52	3	4599	5928	4072	5144	0.07	0.002	0.72
37	TAG	52	4	2149	4450	1662	4058	0.12	<0.001	0.86
38	TAG	52	5	420	1215	419	964	0.12	<0.001	0.12
39	TAG	52	6	51	201	91	184	0.64	<0.001	0.24
40	TAG	54	2	600	537	380	329	<0.001	0.17	0.88
41	TAG	54	3	1529	1677	2265	1322	0.19	0.008	<0.001
42	TAG	54	4	1255	2259	1567	2044	0.75	<0.001	0.09
43	TAG	54	5	765	2328	948	2433	0.32	<0.001	0.79
44	TAG	54	6	252	1496	453	1814	0.007	<0.001	0.53
45	TAG	56	2	88	41	20	26	<0.001	<0.001	<0.001
46	TAG	56	3	229	211	233	128	0.12	0.02	0.09
47	TAG	56	4	154	305	213	251	0.9	<0.001	0.006
48	TAG	56	5	129	529	374	580	<0.001	<0.001	0.002
49	TAG	56	6	123	812	514	1041	<0.001	<0.001	0.14
50	TAG	56	7	104	1038	539	1479	<0.001	<0.001	0.98
51	TAG	56	8	52	757	251	1234	<0.001	<0.001	0.06
52	TAG	56	9	8	123	43	182	0.003	<0.001	0.42
53	TAG	58	2	9	4	2	2	<0.001	<0.001	<0.001
54	TAG	58	3	31	13	9	8	<0.001	<0.001	0.004
55	TAG	58	4	21	31	20	20	0.11	0.2	0.19
56	TAG	58	5	15	39	39	36	0.04	0.04	0.01
57	TAG	58	6	22	84	78	110	<0.001	<0.001	0.11
58	TAG	58	7	27	162	129	254	<0.001	<0.001	0.77
59	TAG	58	8	26	226	167	369	<0.001	<0.001	0.98
60	TAG	58	9	18	235	99	455	<0.001	<0.001	0.01
61	LPA	18	0	4	6	7	10	<0.001	<0.001	0.13
62	PA	36	2	10	6	11	8	0.55	0.16	0.68
63	PA	36	3	20	22	23	27	0.12	0.25	0.52
64	PA	36	4	8	13	6	19	0.08	<0.001	0.004
65	PA	38	4	89	111	142	236	<0.001	0.001	0.04
66	PA	38	5	20	20	23	25	0.07	0.6	0.66
67	PA	38	6	8	9	12	16	<0.001	0.07	0.3
68	LPC	16	0	1	2	2	2	0.68	0.09	0.4
69	PC	32	0	52	42	54	62	0.06	0.84	0.14
70	PC	34	1	574	315	990	435	0.003	<0.001	0.08
71	PC	34	2	639	753	715	1018	0.08	0.03	0.33
72	PC	34	3	20	17	18	26	0.01	0.53	0.85
73	PC	36	2	433	586	382	519	0.32	0.02	0.89
74	PC	36	3	204	143	225	94	0.64	0.002	0.24
75	PC	36	4	548	561	645	713	0.09	0.58	0.71
76	PC	38	3	98	123	129	79	0.69	0.45	0.03
77	PC	38	4	368	534	325	657	0.55	<0.001	0.22
78	PC	38	5	101	86	157	114	0.01	0.08	0.37
79	PC	38	6	236	338	279	496	0.05	0.003	0.26
80	PC	40	4	11	11	11	12	0.9	0.67	0.71
81	PC	40	6	86	144	87	200	0.19	<0.001	0.21
82	PC	40	7	40	42	55	51	0.11	0.87	0.62
83	PC	40	8	11	12	17	13	0.12	0.58	0.37
84	PC	42	6	1	2	0	2	0.02	<0.001	0.75
85	LPE	16	0	27	18	43	39	0.01	0.35	0.67
86	LPE	18	0	42	50	51	78	0.16	0.18	0.44
87	LPE	18	1	17	14	32	21	0.04	0.2	0.43
88	LPE	20	0	60	75	55	84	0.87	0.01	0.41
89	LPE	20	1	13	7	23	9	0.004	<0.001	0.03
90	LPE	20	2	5	5	5	8	0.4	0.38	0.4
91	PE	34	1	133	63	283	102	<0.001	<0.001	0.04
92	PE	34	2	259	300	377	598	0.001	0.03	0.13
93	PE	36	1	389	210	664	289	0.005	<0.001	0.1
94	PE	36	2	336	679	425	714	0.38	<0.001	0.7
95	PE	36	3	313	439	510	492	0.05	0.39	0.25
96	PE	36	4	908	623	1563	1004	0.002	0.007	0.37
97	PE	38	1	88	74	129	74	0.1	0.008	0.1
98	PE	38	2	400	544	356	465	0.25	0.02	0.75
99	PE	38	3	295	281	379	288	0.32	0.26	0.41
100	PE	38	4	1737	2209	2108	2772	0.09	0.04	0.73
101	PE	38	5	687	771	1198	1046	0.003	0.79	0.36
102	PE	38	6	1068	1396	1693	2497	0.002	0.03	0.35
103	PE	40	4	352	478	284	538	0.94	0.003	0.29
104	PE	40	6	632	1059	799	1625	0.02	<0.001	0.19
105	PE	40	7	291	346	548	657	<0.001	0.25	0.7
106	LPI	18	0	26	31	52	78	0.001	0.15	0.32
107	PI	34	1	98	15406	136	220	0.06	0.01	0.59
108	PI	34	2	62	78	74	131	0.04	0.03	0.2
109	PI	36	2	102	120	64	152	0.84	0.003	0.04
110	PI	36	4	331	249	614	335	0.001	0.002	0.07
111	PI	38	3	529	810	718	511	0.53	0.68	0.008
112	PI	38	5	154	108	480	141	<0.001	<0.001	<0.001
113	PI	38	6	19	16	21	22	0.24	0.77	0.55
114	PI	40	4	57	34	75	36	0.16	<0.001	0.27
115	PI	40	5	94	29	122	39	0.12	<0.001	0.48
116	PI	40	6	38	37	56	48	0.22	0.7	0.74
117	LPS	18	0	4	4	6	8	0.002	0.31	0.16
118	PS	36	4	70	43	103	59	0.006	<0.001	0.35
119	PS	38	4	488	632	792	1299	<0.001	0.001	0.06
120	PS	38	5	16	7	42	10	<0.001	<0.001	<0.001
121	PS	38	6	48	57	71	78	0.02	0.39	0.94
122	PS	40	4	20	22	25	39	0.001	0.02	0.08
123	PS	40	6	196	326	277	442	0.05	0.005	0.72
124	PG	34	1	203	129	255	170	0.04	<0.001	0.79
125	PG	34	2	79	35	20	30	<0.001	0.001	<0.001
126	PG	38	4	18	13	8	16	0.09	0.36	0.002
127	PG	38	6	5	9	4	12	0.61	<0.001	0.04
128	PG	42	10	5	8	3	15	0.02	<0.001	<0.001

All values are sums of individual lipid species and expressed in peak area × 10^3^. FFA: free fatty acid; MAG: monoacylglycerol; DAG: diacylglycerol; TAG: triacylglycerol; LPA: lysophosphatidic acid; PA: phosphatidic acid; LPC: lysophosphatidylcholine; PC: phosphatidylcholine; LPE: lysophosphatidylethanolamine; PE: phosphatidylethanolamine; LPI: lysophosphatidylinositol; PI: phosphatidylinositol; LPS: lysophosphatidylserine; PS: phosphatidylserine; PG: phosphatidylglycerol; SM: Sphingomyelin. Analysis was performed using the method of least squares to fit general linear models in SAS.
